# Preconditions for nurses' perceived organizational support in healthcare: a qualitative explorative study

**DOI:** 10.1108/JHOM-03-2020-0091

**Published:** 2021-09-13

**Authors:** Christian Gadolin, Maria Skyvell Nilsson, Axel Ros, Marianne Törner

**Affiliations:** Department of Health Sciences, University West , Trollhättan, Sweden; Region Jönköping County and Jönköping Academy for Improvement of Health and Welfare, Jönköping University , Jönköping, Sweden; School of Public Health and Community Medicine, Institute of Medicine, University of Gothenburg , Gothenburg, Sweden

**Keywords:** Nurses' health, Healthcare quality, Healthcare efficiency, Management support, Nurses' working conditions, Healthcare staff retention

## Abstract

**Purpose:**

The purpose of this paper is to inductively explore the context-specific preconditions for nurses' perceived organizational support (POS) in healthcare organizations.

**Design/methodology/approach:**

A qualitative interview study was performed, based on the critical incident technique (CIT), with 24 registered nurses in different specialities of hospital care.

**Findings:**

The nurses perceived three actors as essential for their POS: the first-line manager, the overarching organization and their college. The preconditions affecting the nurses’ perceptions of organizational support were supportive structuring and structures at work, as well as individual recognition and professional acknowledgement.

**Originality/value:**

Previous studies of POS have mostly had a quantitative outset. In this paper, context-specific preconditions for nurses' POS are described in depth, enabled by the qualitative approach of the study. The findings may be used to guide healthcare organizations and managers aiming to foster nurses' POS, and thereby, benefit nurses' well-being and retention, as well as healthcare quality and efficiency.

## Introduction

Ensuring high-quality care with optimal resource utilization is a fundamental principle of healthcare, for ethical and economic reasons ( Gadolin, 2017 ). Since healthcare work is staff intensive, the health and well-being of professional healthcare employees are a cornerstone for achieving this ( Demerouti *et al.* , 2001 ; Eklöf *et al.* , 2014 ). The internationally recognized increase in poor mental health among healthcare employees ( Bridgeman *et al.* , 2018 ; O’ Keeffe *et al.* , 2015 ), and not least among nurses, thus implies a major challenge for ensuring efficient delivery of high-quality healthcare services. Previous research has found that nurses often perceive high levels of stress, symptoms of burnout and low levels of job satisfaction, all of which imply major challenges for nurse recruitment and retention ( Aamir and Hamid, 2016 ; Almada *et al.* , 2004 ; Johnson *et al.* , 2016 ; Weber, 2010 ; Whitehead *et al.* , 2015 ). In concordance with this, multiple studies have established high nurse turnover in developed and developing countries alike, often closely related to job dissatisfaction ( Lu *et al.* , 2019 ). In continuously striving for high-quality, efficient healthcare, it is therefore essential for healthcare organizations to better ensure nurses' mental health and job satisfaction ( Eklöf *et al.* , 2014 ; Pousette *et al.* , 2017 ). Since a large body of research shows perceived organizational support (POS) ( Eisenberger *et al.* , 1986 ) to be related to, for example, employee job satisfaction and well-being, work engagement and performance, and reduced withdrawal behaviour ( Kurtessis *et al.* , 2017 ), strengthening nurses' POS appears to be a viable route in this direction.

Many quantitative studies have been performed on POS antecedents and outcomes ( Ahmed *et al.* , 2015 ; Kurtessis *et al.* , 2017 ; Riggle *et al.* , 2009 ), but contextualized and qualitative studies are lacking. Such studies can complement quantitative approaches by deepening the understanding of POS development and inductively delineating its particular requirements in specific contexts. While the benefits of achieving high POS among employees are well supported in previous research, the mechanisms for attaining POS deserve more research attention. Meta-studies of quantitative research have identified some general, basic antecedents associated with POS, such as fair organizational procedures, supervisory and co-worker support, and growth opportunities ( Ahmed *et al.* , 2015 ; Kurtessis *et al.* , 2017 ). However, POS antecedents are presumably a complex, contextually entangled matter, in dire need of further elaboration and nuance. Such knowledge could help managers at different organizational levels to develop the specific conditions that benefit employees' POS in different occupational settings. While this has previously been implied ( Paul and Phua, 2011 ; Rhoades and Eisenberger, 2002 ), Ahmed *et al.* (2015) makes explicit that preconditions for POS appear, at least to a certain extent, to be sector and organization specific. It also appears plausible that distinct professional groups, within the same sector, require preconditions for high POS that are specific to their work roles ( Riggle *et al.* , 2009 ). Healthcare organizations and managers that provide conditions that strengthen nurses' POS will promote nurses' job satisfaction and mental health, and thus facilitate efficient resource utilization. To provide concrete and contextualized knowledge of what constitutes such conditions, the present qualitative study aims to inductively explore the specific preconditions of high POS among registered nurses in hospital care.

### Theoretical background

POS is defined as employees' “global beliefs concerning the extent to which the organization values their contribution and cares about their well-being” ( Eisenberger *et al.* , 1986 , p. 501). The theory of the beneficial effects of POS is based on the ability of such support to fulfil profound human socio-emotional needs of self-respect, social status and meaningfulness ( Rhoades and Eisenberger, 2002 ; Shanock and Eisenberger, 2006 ). POS theory also postulates, based on a social exchange approach ( Blau, 1986 ), that POS increases an employee's efforts to reciprocate perceived support by striving to meet the organization's goals and helping to achieve desirable organizational outcomes. According to theory, employees tend to interpret organizational actors' actions as representing the organization's intent, rather than its actors' personal motives. This induces social exchange between the employee and the organization ( Eisenberger *et al.* , 1986 ). Several meta-analyses have validated these basic assumptions regarding POS, finding that it leads to the anticipated favourable outcomes for both the organization and its employees. These outcomes include affective commitment to the organization, employee engagement, increased employee performance, job satisfaction and well-being, and reduced withdrawal behaviour ( Ahmed *et al.* , 2015 ; Kurtessis *et al.* , 2017 ; Rhoades and Eisenberger, 2002 ; Riggle *et al.* , 2009 ).

## Methods

To capture the context-specific preconditions for nurses' POS, this study applied an inductive qualitative design, revisiting the two basics facets of POS: the organization's care for its employees' well-being and its valuation of their contribution. We performed in-depth interviews with 24 registered nurses in hospital care (see Table 1 ). The nurses were selected through a process inspired by maximum purposeful variation sampling ( Hammarberg *et al.* , 2016 ; Patton, 2002 ) to obtain rich data with a broad spectrum of personal and contextual conditions. The targeted interviewees were registered nurses working within 15 different care specialities at seven hospitals providing secondary and tertiary care, across two regional organizations in public healthcare. The nurses also varied in terms of job experience. Ethics approval (no. 264-18) for the study was obtained from the Regional Ethics Review Board, Gothenburg.

The interviews were performed according to the critical incident technique (CIT) ( Flanagan, 1954 ). This technique limits the influence of interviewer subjectivity and preconceived notions of interviewee responses. The CIT also enables the interviewees to have better access to context-specific memories and personal experiences of situations relevant to the subject matter ( Wheeler *et al.* , 1997 ). Before the interview, all participants were informed about the aim and procedure of the study and about the right to withdraw from the study at any time. They all provided their written consent to participate. The interviews took place in secluded meeting rooms at the interviewees' respective workplaces. The first and second author, both with substantial experience in qualitative research in healthcare, performed the interviews. Initially, they each performed two pilot interviews, to test the interview guide and ensure inter-researcher alignment in performing the interviews. The pilot interviews were recorded, transcribed verbatim, and read and discussed by the entire research team. Both the interview guide and inter-researcher alignment were found acceptable, so the pilot interviews were included in the further analyses. Before the interviews took place, the interviewees were instructed to reflect upon four situations they had experienced: two situations in which they had/had not perceived that the organization cared for their well-being and two situations in which they had/had not perceived that the organization valued their contribution. During the interviews, the interviewees were asked to recall these situations and retell them as accurately, fully and specifically as possible. The interviewer's role was to encourage and guide the interviewee to elaborate upon the recalled situations. Follow-up questions were posed, but without the interviewers introducing any themes of their own. The interviews were 25–50 min in length. All interviews were transcribed, which resulted in 485 pages of raw data.

The qualitative data analysis was conducted in accordance with the three steps suggested by Miles and Huberman (1994) : (1) data reduction, (2) data display and (3) drawing conclusions and verifying the results. For the first two analytical steps, 16 interviews (additional to the pilot interviews) were performed. Step one was initialized by all the authors reading the transcripts and making detailed individual notes of their initial understanding of the data. These notes were discussed in the research group. This discussion resulted in identifying the nurses' diverse understanding of the organization as an important aspect forming the context-specific preconditions for their POS. As a result, two tentative subject areas were identified: (I) nurses' view of the organization and (II) nurses' context-specific preconditions for POS. Data relevant to each tentative subject area were sorted into nodes by the first and the second authors, using the software NVivo. Within the first tentative subject area, three separate actors were identified as significant for the development of POS (see Table 2 ).

Within the second tentative subject area, the first and second authors collaborated on sorting all the data relevant into the preconditions that foster and hinder, respectively, the nurses' perceptions that the organization cares about their well-being and values their contribution. During this reading and sorting, the two authors extensively discussed the material and the association between identified nodes, as well as their interconnectedness. This resulted in tentative categories based on similarities found in the empirical data as represented in the sorted nodes. Following the forming of the tentative categories, all authors discussed them and their relevance and ensured that all aspects identified in the transcripts were included. In the second analytical step, the first and second authors refined the tentative categories into specific subthemes and themes reflecting the interpretation of their content and relations. This step facilitated the presentability and comprehensibility of the empirical data, which often is underscored as vital when conducting qualitative content analysis (e.g., Ahrens and Chapman, 2006 ; Noble and Smith, 2014 ; Willig, 2014 ). In the third analytical step, the final four of the 24 interviews were performed and analysed with a special aim to verify the validity of the themes and subthemes and ensure the saturation of the data. As these interviews accorded with the results of the previous analysis and presented no new aspects relevant for nurses' high POS, these interviews established the validity of the themes and subthemes and ensured that saturation of data had been achieved. The analysis process of subject area II is illustrated in Table 3 .

### Ethical considerations

The study was performed in accordance with all requirements of the Regional Ethics Review Board, for example, regarding acquisition of written informed consent from the participants and securing safe storage of data and code keys. Only the two interviewing researchers had first-hand knowledge of the individuals involved in the study. By assigning a code to each interview, and transcribing and analysing all interviews in such coded form, confidentiality during the research process was protected. In addition, the risk of identifying participants in the results has been carefully considered by the research group, and specific information about wards, hospitals or situations that could be linked to individuals has been omitted.

## Results

The analysis showed that the nurses mainly perceived three types of actors as significant for the development of POS: the *first-line manager* , who was their direct superior in the organizational hierarchy; the *overarching organization* , which encompassed middle and upper management; and the *college* , which primarily included their immediate colleagues but also those in other departments and wards. However, the nurses did not perceive that these actors acted in isolation from one another.

The three types of organizational actors were the sources of the preconditions for nurses' POS. These preconditions could be categorized into two themes, with two subthemes each, that are briefly summarized in Table 4 .

The analysis also showed that preconditions for the two aspects of POS were empirically intertwined. Acts of the organization generally affected the nurses' perceptions regarding both the organization's care for their well-being and its valuation of their contribution. Therefore, the preconditions presented thematically below generally incorporate both these aspects. However, certain preconditions were understood as affecting primarily one of these aspects. In such cases, this is explicitly pointed out. In the text below, direct quotes are presented to illustrate the qualitative interpretations.

### An organization perceived as three separate, yet intertwined, actors

*The first-line manager* was a primary organizational actor, perceived as responsible for multiple preconditions that affected the nurses' perceptions of how the organization cared for their well-being and valued their contribution.

*The overarching organization* , encompassing middle and upper management, was also perceived as an important actor regarding POS. However, while most nurses expressed trust in their first-line managers, and a belief that they cared about the nurses' well-being and valued their contributions, the same notion was rarely expressed in relation to the overarching organization. The nurses sometimes even perceived that their first-line managers had to defend their interests in relation to the overarching organization, implying an “us versus them” mentality. The overarching organization was often understood as too occupied with managing the whole organization (the hospital), or the entire regional healthcare sector, to be aware of the nurses' contribution and well-being. Therefore, it was unable to express its appreciation and concern for the work a specific ward/department undertook. The nurses' POS was negatively affected when they felt that the overarching organization was not aware of the work they were doing, so not showing appreciation for it. One of the nurses expressed:

I believe that this organization is too large. There is too large a gap between those of us who actually care for the patient and the hospital director and governing bodies. I do not think anyone who has not worked within healthcare can understand how difficult and tearing it is to work as a nurse. It is sometimes horrible, but it is also the most kind and caring thing one can do. It is, however, not a calling. It is a profession and like all other professions, we deserve to be appreciated for the work we do.

*The college* , manifested through social interactions primarily with the immediate colleagues, but also those in other departments and wards, was also seen as an important actor in the organization. It also affected the preconditions of nurses' perceptions of how the organization cared for their well-being and valued their contributions. Many of the situations that the nurses described as positively or negatively affecting POS included interactions with colleagues.

### Theme 1: organizational structuring and structures supporting professional work and well-being

This theme included the two subthemes of *structuring* the work and work *structures* . These were, by far, the most common subthemes expressed by the interviewees. The two subthemes, described in more detail below, were largely integrated, although in some situations one subtheme was more important than the other. Both aspects were organizationally related preconditions, beyond the direct control of the individual nurse and affected the nurses' ability to perform their work.

#### Structuring that supports professional work and well-being

Temporary and ad hoc organizational structuring is often required in healthcare organizations. Such structuring incorporates fluid, instant and spontaneous actions of organizational representatives that shape the conditions that influence the nurses' ability to perform their job. Structuring was often described as rearranging tasks among the staff and managing sufficient staff requirements. Most nurses stated that a first-line manager who could solve structural challenges in a supportive way for all involved both safeguarded the nurses' well-being and showed valuation of their contributions. However, when the organization underwent too much structuring too often, and such ad hoc structuring was systematically implemented, this negatively affected the nurses' ability to maintain high-quality care and/or their own well-being and hence decreased their perceptions of organizational support. The nurses also described several situations in which the first-line manager was unable to handle structural demands in a supportive way. Such non-supportive structuring focused on maintaining healthcare production, neither considering the nurses' ability to carry out high-quality care nor safeguarding their health and well-being. These situations were often related to ordinary tasks that were to be performed without the ability to allocate adequate time, either for performing the task or for recovery. These types of structuring often resulted in the nurses experiencing symptoms of burnout and sometimes seeking other employment. When reflecting upon the perceived conflict between organizational structuring and individual needs, one nurse stated:

There have been several situations where I have felt that I am not a priority at all; it is always the delivery of care that is. And I understand that it must be prioritized – but I still want them [the organization] to take my individual wants and needs into consideration. I do not want to be perceived solely as one of many faceless drones within the organization, intended only to perform certain tasks and fulfil a specific function. Ensuring my well-being at work ought to be imperative for them [the organization].

The nurses presented various arguments regarding which organizational actor should be held accountable for such insufficiencies. Some nurses placed full responsibility on the first-line manager's inability to affect structuring, leading to patient overcrowding or staff shortage. Others argued that the first-line manager did not have authority to affect organizational structuring, so placed the responsibility on the overarching organization. Still other nurses highlighted their own responsibility and attributed the unsatisfactory situation to their own lack of skills, experience and ability to provide high-quality care at a high pace.

Structural demands that were not solved in a supportive way were frequently described in the interviews and could indicate a lack of more elaborate and long-term structures and planning in the organizing of patient care. The value of such elaborate and sustained structures is described in the next subtheme.

#### Work structures that support professional work and well-being

This subtheme describes the importance of elaborate, sustained organizational work structures and how they contribute to the nurses' perception of an organization that cares for their well-being and values their contributions. Such structures contributed to their ability to carry out high-quality care, while also maintaining their own health and well-being. Work structures represented rigid, even institutionalized, characteristics of the organization that affected the nurses in their everyday exercise of their profession. One nurse described the importance of supportive work structures and how they were developed:

Our ward was previously very much disorganized. We had a structure that counteracted patient safety, due to the sheer number of tasks each nurse had to manage. As such, we did not have the ability to perform each task adequately. But then we reorganized our work. The reorganization was initiated by our manager, but we [nurses] were always included in it. The result of the reorganization is that we currently have a much better continuity with the patients we care for and are able to provide higher quality care. The reorganization has resulted in being able to do a much better job than previous structures allowed us to do.

However, the nurses' more often described situations in which work structures were deemed inapt, or even lacking, which negatively affected how they perceived organizational concern for their well-being and valuation of their contribution. In one such example, the nurses themselves were expected to call in staff in times of shortage, while still doing their regular work. Several nurses expressed that they were expected to resolve such situations to the best of their ability. As a consequence, they perceived that functional structures to control staffing were lacking. This led to the perception that the organization was not attentive to, or did not care about, their individual job situations, but only considered the organization's ability to continue operating.

The nurses often perceived that it was up to them to solve the challenges stemming from lack of foresight in scheduling. Such challenges often emanated from the policy of allowing the nurses to influence their own work schedules. Some nurses, often with considerable job experience, stated that this freedom was beneficial and a manifestation of how the organization cared for their well-being. This scheduling flexibility allowed them to consider leisure activities and family life. On the other hand, some nurses perceived that the lack of clear structure regarding scheduling had negative consequences. These consequences often entailed dilemmas, such as the nurses' responsibility to offer continuous, high-quality care juxtaposed with their, and their colleagues', need for sufficient time to recover between shifts and have a functional social and family life. When commenting on the function of supportive work structures, one nurse stated:

It makes me feel calm and secure, and enables me to focus on being a nurse. I don’t have to go to work being irritated and frustrated at structures or the organization itself.

### Theme 2: individual recognition and professional acknowledgement

Theme 2 is related to nurses' social needs, both as individuals and professionals. This theme presents the preconditions that satisfy a desire to be seen and recognized both as an individual and in the role as a nurse. In other words, the nurses wanted their personal situation and their professional experience, competence and efforts taken into account and acknowledged and utilized adequately in their daily work.

#### To be seen and recognized

An important subtheme related to situations in which the nurses perceived themselves as being seen and recognized by their first-line managers and colleagues. This subtheme included every day, sometimes seemingly trivial, communication, such as greeting the nurses when they arrived at work. It also included the first-line managers explicitly recognizing the nature of the work the nurses performed, by showing interest in their performance and giving feedback. When the managers engaged in such communication, it positively affected the nurses' POS. Nurses' POS was negatively affected if such basic communication was lacking, or managers failed to acknowledge the nurses' working conditions, especially excessive workloads. Even simple tokens of appreciation, such as a bowl of candy when working overtime, were important signs of recognition for the nurses and the work they did.

Nurses' perception of organizational support, particularly in relation to care for their well-being, was positively influenced when the first-line managers accommodated individual nurses' requests and needs. This could relate to situations in which the nurses perceived that the first-line managers tried to satisfy individual needs and requests related to their work, but more often, to their private lives. This involved a broad range of individual adaptations of the work situation in connection to, for example, sick leave, pregnancy and family members' health. Other adaptations involved the introduction of equipment or computer accessories to reduce physical workload or pain. In most cases, the nurses' said that their first-line managers did their best to meet such needs and requests. However, there were also perceptions that the organization did not care for the nurses' well-being. One nurse gave an example:

Not that long ago, I spoke to my boss. I was not feeling well because of my night work. I have been working nights for a long time, but recently felt it wasn’t good for me, anymore, because I hardly slept at all. When I spoke to my boss about it, she demanded a medical note, which I got and gave to her. However, the situation upset me, because I felt I was not treated well. It felt like she perceived it to be very demanding when I gave her the medical note. She said, “More and more of you nurses have pointed out that you are unable to work nights. It will end up in that many of you will have to find other jobs.” I was really upset, because there is no other job that I want to do. I really like this job. So in the end, I will end up saying, “Okay, I will work nights again,” because I want to keep this job.

The nurses also described the importance of being seen and recognized by colleagues. Being appreciated and acknowledged for their skill and expertise, and receiving constructive and relevant feedback from their colleagues were important components in perceiving support from the organization. The situations that most of the nurses identified as supportive interactions with colleagues entailed interactions with other nurses, although interactions with other healthcare professionals were also sometimes identified as supportive. Descriptions of interactions, when the nurses perceived the organization as not caring, mostly involved physicians or colleagues who were not on their immediate care team. In such interactions, the nurses received criticism for not performing certain tasks in alignment with their colleagues' wishes and requirements. These negative interactions often involved tasks the nurses rarely performed. These situations were often attributed to a lack of communication between different wards and departments in the organization.

#### Valuation and utilization of competences

Nurses' perception of support was influenced by the valuation and utilization of their competences related to their role as a nurse and their personal knowledge and experience. When the nurses perceived their competences to be valued by the organizational actors, and appropriately utilized, this positively influenced their POS. Many nurses recalled situations in which they experienced such support. However, if they felt that the organization did not value or appropriately utilize their specific competences, the nurses' POS was negatively affected. Such situations could sometimes occur when they openly expressed their dissatisfaction. When recalling such a situation, one nurse said:

When I told my manager that none of the nurses wants to continue working at this department due to the infeasible workload, the manager simply replied that “There are none of you [nurses] who are invaluable nor irreplaceable.”

Situations in which the nurses were required to perform tasks or procedures for which they did not consider themselves to have the appropriate competence or experience also negatively influenced their experience of support. Experiences of such competence deficits occurred when the nurses were expected to perform complex medical interventions without sufficient training and expertise. Other situations in which the nurses perceived that their competence was not properly utilized included being ordered to perform tasks and procedures not inherent to their role or function as registered nurses. Being told to perform tasks normally performed by an orderly were examples of such situations, which often occurred due to staff shortages. All these situations led the nurses' to perceive that the organization did not respect their specific competence. This negatively affected their perceptions of the organization's valuation of their contribution.

The nurses' perceptions of organizational support were positively influenced by the first-line managers' support for their professional development and extended responsibility. In general, the nurses expressed that they had ample opportunity to develop their professional skills, through courses and further training. Many also stated that they had the opportunity to use and develop their competence by taking responsibility for specific task areas in their ward/department. Supervising nursing students, or monitoring improvement of work in relation to specific tasks and procedures such as patient safety, were examples of this. The nurses who engaged in such activities perceived it to be an expression of the organization's valuation of their contribution, as well as its care for their well-being.

The nurses who perceived that their first-line managers did not allow them to develop their professional competences, and/or did not support them in taking on extended responsibility, saw this as an expression of the organization caring neither for their well-being nor for their contribution. The same line of reasoning was found among nurses who perceived that their first-line managers did not encourage or commend such activities. Most commonly, nurses took the initiative to engage in such activities. First-line managers' support for these initiatives was understood as acknowledgement and appreciation for specific individuals' contributions to the organization. When expressing their feelings about not being allowed to take part in a certain educational course, one of the nurses stated:

I would have been tremendously disappointed if I were not allowed to take this specific course. I have been working here for two years now, and I haven’t been able to take part in any other courses previously.

Salary was also described as relevant in relation to the organization's valuation of nurses' contribution. The most common critique was that nurses' salaries were low in relation to the content of their jobs. The nurses often made comparisons to equivalent jobs with higher salaries (at private companies or in elderly care organized by the local municipalities). The nurses were concerned about the lack of forums and possibilities for discussing salary with their superiors. They often perceived a weak, sometimes even non-existent, relationship between their individual job performance and their salary progression. This contributed to the perception that the organization did not value their work contributions. Sometimes they attributed this lack to the inability of the first-line managers to actually influence their salaries, due to strong budget constraints. One nurse cited the response from the first-line manager when the subject of salary was discussed at an annual individual performance follow-up meeting: “There is no use that you mention figures. You know that we do not discuss salaries [in this organization].” Other nurses attributed the lack of salary discussions to the sometimes still-prevailing understanding of nursing as a calling, rather than a profession – the notion that the content of the work itself should be reward enough and monetary compensation of less importance. Such a view lowered the acceptability of discussing salary. The nurses, who perceived that they were unable to influence their salaries, or even meaningfully to discuss the matter with their first-line managers based on their job performance, experienced that the organization did not value their contribution.

## Discussion

Previous research has convincingly shown that POS supports a range of desirable organizational outcomes, such as affective commitment to the organization, employee engagement and increased employee performance and retention ( Ahmed *et al.* , 2015 ; Kurtessis *et al.* , 2017 ; Rhoades and Eisenberger, 2002 ; Riggle *et al.* , 2009 ). This indicates that strengthening nurses' POS can be a viable route for ensuring quality and efficiency of healthcare services through attaining higher job satisfaction and health and reduced withdrawal behaviours. This paper contributes with in-depth and contextualized understanding of how to support nurses' POS.

The analysis showed that the nurses perceived that three distinct organizational actors influenced their POS: the first-line manager, the overarching organization and the college. Whereas the first-line manager was distinctly related to a particular individual (a nurse's immediate superior), the overarching organization comprised multiple individuals (mid- and upper-level managers). This was also the case regarding the college, which included not only other nurses but also healthcare workers in other professions. Designating and delineating the actors whom the nurses perceived to influence their perceptions of organizational support is an important contribution, as it facilitates organizational development to support specific actors' ability to foster nurses' POS. Identifying supervisors and co-workers as important actors in relation to POS is concordant with previous studies ( Kurtessis *et al.* , 2017 ). In the context of healthcare organizations, ascribing the importance of interactions with colleagues as a precondition for POS can be understood in relation to the professionalization of the nursing occupation ( Beedholm and Frederiksen, 2015 ; Råholm *et al.* , 2010 ), which entails that collegiality often becomes essential for performing nursing work (cf. Freidson, 1986 ; Freidson, 2001 ).

Our results suggested that acts of the overarching organization are important for POS. However, while the nurses often maintained distinct perceptions regarding their first-line managers, their apprehension about the overarching organizations was less well defined but also generally more negative. Whereas the first-line manager often was perceived as protective of nurses' well-being and valuing their contribution, the overarching organization was often not seen in the same light. This finding is interesting in relation to the definition of POS that states, “[E]mployees develop global beliefs concerning the extent to which the organization values their contributions and cares about their well-being” ( Eisenberger *et al.* , 1986 , p. 501). Such an assumption entails that different organizational actors are interpreted uniformly. The results of the present study instead indicate a need to study how different actors influence POS, and which types of actions by different actors are essential for developing POS. This is in line with previous research on organizational safety climate regarding both patient and staff safety in healthcare ( Pousette *et al.* , 2017 ), as well as in other occupational branches ( Zohar, 2008 ). It indicates that the organization as represented by the proximate environment (the close colleagues and the first-line manager) may be interpreted quite differently to the more distal parts of the organization, and these should therefore be studied as different entities (cf. Kines *et al.* , 2011 ). The substantial organizational complexity in healthcare organizations, with multiple actors and norms ( Andersson and Gadolin, 2020 ), may further hinder formation of global beliefs regarding the organization. This emphasizes the particular need to study the specific contribution to employee POS at different organizational levels in this sector. In the present study, the nurses more often specified particular actions among their first-line managers and their colleagues that influenced their POS. This is in alignment with previous findings that some organizational actors more closely embody the organization than do others ( Eisenberger *et al.* , 2010 ). However, the nurses also acknowledged the fundamental role of upper management in relation to POS. Yet, they were less able to describe specific actions at this organizational level. This vagueness about upper management may indicate a serious problem, since its influence on POS was generally interpreted as negative.

It is noteworthy that social interaction with colleagues seems to be vitally important for the nurses to receive individual recognition and to strengthen their perceptions of being able to significantly contribute to organizational goals and therefore to increase their POS. This aligns well with results from a study in safety-critical industries, which suggested that social support and trust from co-workers validated the individuals' sense of competency. This sense of competency was the first defence in dealing with work ambiguity, but it also anchored basic work identity ( Jackson, 2017 ). It is also in concordance with previous studies in healthcare, indicating the importance of good relations within and between professions to attain staff health and safety, and patient safety ( Eklöf *et al.* , 2014 ), as well as general quality developments in healthcare settings ( Gadolin and Andersson, 2017 ; Gadolin, 2018 ). Explicitly highlighting the importance of interactions with colleagues for the nurses' POS is an important contribution of this paper.

The important context-specific preconditions for the nurses' POS were found to separate into two distinct themes. The first theme incorporated the aspects of supporting work structuring and structures that facilitate the nurses' professional work. Whereas structuring incorporated more immediate and short-term acts of representatives of the organization, organizational structures encompassed more sustained characteristics of the organization that affected the nurses' ability to carry out their work. Supportive work structuring and structures entailed the nurses perceiving that their well-being was safeguarded and enabled the nurses to focus on their main task of providing care. Both work structuring and structures positively affected the nurses' POS if they were perceived as facilitating the nurses' ability to provide high-quality care while simultaneously safeguarding their well-being. If their supervisors were solely perceived as prioritizing the production of care, the nurses' POS declined. These findings are aligned with previously affirmed preconditions affecting employees' POS (see Kurtessis *et al.* , 2017 ), especially those that underline the necessity of value congruence between employees and employers (e.g., Edwards and Cable, 2009 ; Erdogan *et al.* , 2004 ; Hoffman *et al.* , 2011 ). This was further shown through the negative impact on the nurses' POS when they perceived that management did not prioritize high-quality care. The findings also highlight and explicate how supportive work structuring and structures, and the contrary, may manifest in healthcare organizations. Whereas the nurses had somewhat different perceptions regarding the responsibility of different organizational actors for structuring their work and sometimes attributed flaws to own inability to organize their work, the first-line manager and the overarching organization were generally deemed responsible.

The second theme found to be of importance for the nurses' POS related to individual recognition and professional acknowledgement through interaction with organizational actors. The nurses' POS was affected positively if they felt that they were seen and recognized, that their individual and specific competences were valued and utilized properly, that they were able to develop professional competences and attain extended responsibility, that their individual requests and needs were accommodated and that their salary level was influenced by their individual contribution at work. The necessity of being seen and recognized at work is underscored by the fact that POS is driven by socio-emotional needs of self-respect and social status ( Eisenberger *et al.* , 1986 ; Rhoades and Eisenberger, 2002 ; Shanock and Eisenberger, 2006 ). The finding that nurses perceived that their ability to develop their professional competences and attain more responsibility influenced their POS aligns with the professionalization of the nursing field ( Beedholm and Frederiksen, 2015 ; Råholm *et al.* , 2010 ). It is also in concordance with previous findings showing that developmental opportunities are associated with high POS ( Kurtessis *et al.* , 2017 ). The importance of the nurses' perception of fair wages affecting their POS is also supported by previous research, especially studies highlighting the effects of perceived organizational justice ( Bell and Khoury, 2016 ; Colquitt *et al.* , 2001 ; Lavelle *et al.* , 2007 ; Moorman *et al.* , 1998 ; Viswesvaran and Ones, 2002 ).

This paper highlights the specific, concrete conditions and actions that are important for nurses' POS in healthcare organizations. In doing so, the paper underscores the importance of managers being able to provide sustained, supportive organizational structures and manage ad hoc structuring, as well as to accommodate nurses' individual and professional needs. Such knowledge can guide organizational development aiming at staff health, as well as quality and efficiency in healthcare. Moreover, it identifies the importance of the first-line managers for nurses' POS. It therefore points to the need for future research to investigate the organizational preconditions for these managers' ability to provide ample conditions for nurses' POS. Further, the results indicate that the overarching organization must convincingly demonstrate its appreciation for the nurses' contributions and care for their well-being. Despite the fact that this qualitative study emanates from a Swedish context, the necessity of safeguarding nurses' health and well-being is a universal phenomenon (cf. Aamir and Hamid, 2016 ; Almada *et al.* , 2004 ; Johnson *et al.* , 2016 ; Weber, 2010 ; Whitehead *et al.* , 2015 ), as is efficient resource utilization ( Gadolin, 2017 ). This indicates that the results of this paper may inform and guide healthcare management in varied national contexts. Further research should, however, be conducted to establish the validity of this deduction. Moreover, the results further strengthen the need for qualitative studies on context-specific preconditions of POS for other staff in healthcare organizations, as well as in other occupational branches.

## Conclusion

This qualitative study among nurses in healthcare aims to revisit the two basics facets of POS: the organization's care for employees' well-being and its valuation of their contribution. Our results suggest that nurses perceive three organizational actors as vitally important for their POS: the first-line manager, the overarching organization and the college. The first-line manager and the overarching organization are essential in assuring supportive structuring and sustained structures at work. Interactions, mainly with the first-line manager and colleagues, were vital for meeting the nurses' need for individual recognition and professional acknowledgement and thus pivotal for high POS. This paper explains the contextual-specific and concrete preconditions for nurses' POS, complementing and contextualizing the quantitative disposition that permeates previous research concerning POS. It also shows how different actions of different healthcare organizational actors in concert may strengthen nurses' POS. Such specific and contextual knowledge of healthcare organizations can help managers to better support nurses' health and well-being, organizational commitment and intention to stay in their current positions. There is also good reason to believe that such a development would strengthen both the quality and efficiency of healthcare. The study identified the importance of the first-line managers, as well as the overarching organization, for nurses' POS. Future research should therefore investigate the organizational preconditions for the first-line managers' ability to provide preconditions that foster nurses' POS and the fundamental role of the overarching organization in this respect.

## Figures and Tables

**Table 1:** Description of the informants

Number of informants (per geographical region)	*N* = 24 (region X: 12, region Y: 12)
Age, years; mean (SD); median (min-max)	36.7 (11.6); 34 (22–65)
Gender	F: 23, M: 1
Experience as a nurse, years; mean (SD); median (min-max)	11.0 (12.0); 8 (0.5–47)
Number with formal specialist nursing education	5
Employment, % of full-time (SD)	95 (14)
Presently engaged in type of healthcare( *n* )	Surgery (5), internal medicine (7), geriatric acute care (1), geriatric open care (1), orthopaedics (2), orthopaedic medicine (1), intensive care (1), infectious diseases (1), maternity care (1), paediatric internal medicine (2), paediatric orthopaedics (1), neonatal care (1)

**Table 2:** Illustration of analysis of subject area I ( *n* = number of interviews where the node was identified, ref = total number of references in the node, *R* = region and *P* = participant code)

Subject area I: Nurses' view of the organization		
*Nodes, illustrated with quotes*		
*Views of the organization (n: 18, ref: 40)* [G]enerally, you can say this, that the organization that is around me, that is, my manager, head of the unit.… If you go beyond this and imagine the big, complex, tricky organization, it feels more anonymous and mysterious, I think. All the people, structure and construction are a mystery to me, how it works.... My boss is good, great, but the others feel a bit threatening almost (R.Y: P.10)	The first-line manager The overarching organization The college	Three separate, yet intertwined actors
*Colleagues/working group (n: 19, ref: 58)* We are an attractive workplace and that is because we are a good bunch as well, and we are the ones who struggle with It [the work]. We don't need managers, to feel good together in the working group.... We do not have the managers to thank and not the organization either, but it is because we as staff have tried to protect those that we like [our colleagues] (R.Y: P.8)		

**Table 3:** Illustration of the analysis of subject area II, including nodes (illustrated with quotes), tentative categories, subthemes and themes ( *n* = number of interviews where the node was identified, ref = total number of references in the node, *R* = region and *P* = participant code)

Subject area II: Nurses' context-specific preconditions for POS			
Nodes, illustrated with quotes	Tentative categories	Subtheme	Theme
*Understanding of the work (n: 6, ref: 7)* There [at a former ward], the first-line manager had a background and had worked in a medical department before. So, I felt that she had more understanding of the situation, and she was very well aware that it was a tough work situation, high workload and limited staff and did the actions she could in that situation.... She comes in and helps. And it is vastly appreciated. She sees that it can be tough. That it does not work always as it is intended (R.X: P.11) *Work ability (n: 8, ref: 11)* When working three shifts, in healthcare in general, sleep and recovery are important. But it becomes a bit paradoxical when the first-line managers say: “You get to work double shifts today because we cannot get any other to do it” (R.Y: P.7) *Work structures (n: 21, ref: 79)* I really didn't want to come [to work], because you knew that now it's a lot and it's not so fun because then you work day and so you have had a lot. Usually it's hard to wind down later [after work]. And then you felt they [managers] didn't care what you say. Then I know they are tied to demands from above, but they did not cut down in-hospital beds.... Yes, and so it was evening and it was really heavy, and after two days. And then I said it again, “This isn’t working; it is too hard.” Then they cut down in-hospital beds, but I felt that it should have been done in the first place (R.X: P.5) At least during some shift in the evening and weekends, when we are fewer, we now have a more experienced nurse to ask. And that's good. There had been some complaints after an evaluation last summer, that there were so many new nurses working together. And it wasn't good. But then the manager made sure that when we set the schedule we made sure that it was an experienced nurse at least every shift. It makes sense, I really think (R.X: P.2)	Recognize the work Recognize and handle the workload A proactive structuring of work	Structuring that supports professional work and well-being Work structures that support professional work and well-being	Organizational structuring and structures supporting professional work and well-being
*To be seen (n: 14, ref: 26)* Our managers say hello every morning. So, like this, “Good morning N.N.” kind of looks at me, “You're going to be here and here” or handing out some alarms and some other things.… I think that is a pretty good start to the day. (R.X: P.1) And if I'm going to think about the organization, my colleagues, I think we are very good at giving appreciation for each other's work.... And then there were many of the colleagues who said that “if it had not been for you, she would not have been allowed to go home” (R.X: P.8) *Feedback (n: 16, ref: 31)* [W]hen I get to work I get called up and they [the first-line managers] say, “You've done a great job.” … When you have salary discussions, then you go through many points, and I think it's pretty nice that the manager does not follow the instructions completely, but that she talks quite freely about my strengths and weaknesses.... But colleagues are better at giving feedback to each other, much more often than from my manager (R.X: P.8) *Individual needs (n: 16, ref: 31)* I'm not my disease. I can do my job. But I think you are very well taken into account, if you have problems and feel worse in periods, and they [first-line manager] try to solve it so that you are still able to work (R.Y: P.12) *Care assignment (n: 6, ref: 10)* It's good that you, or I, are making an effort to make sure that this patient can be admitted. Even if you have a large workload, we still try to look after the patient's best interests. And this is appreciated [by the first-line manager] (R.Y: P.5) *Competence/experience (n: 10, ref: 21)* We're a little short of consultants. It makes them rely very much on the nurses, that she can and knows what to do. And I can stand in situations where I … thus I have the question, but they bounce back “what do you think?” And “how do you usually do?” (R.Y: P.2) *Competence deficit (n: 5, ref: 12)* [T]hen my manager just told me, you could handle this. And I was terrified. I can't stand by and promise to load that machine! Somehow, I stood there in the afternoon and worked that thing out, with the PD nurse down at the reception. So I fixed it all weekend. But, you did not get any appreciation [from the first-line manager] (R.X: P.1) *Professional development and responsibility (n: 14, ref: 25)* I have had the opportunity to step into the ambulance service, even though I am employed here, so I see it as a kind of, what to say, appreciation.... Yes, it's a privilege for me (R.Y: P.4) *Salary (n: 9, ref: 14)* Therefore, you think you want them to show appreciation for everyone who works as a healthcare professional. It should be rewarded at a reasonable level, and the level of the wages, it has not been enough (R.Y: P.6)	Manager and colleagues who pay attention and reconnect Meet up with personal needs Recognize professional engagement Utilize existing competence/experience Ensure competence for the care assignment Opportunities for learning and developed responsibilities Valuation through a fair payment	To be seen and recognized Valuation of competence and its utilization	Individual recognition and professional acknowledgement

**Table 4:** Brief summary of the themes and their respective subthemes

*Theme 1* : Organizational structuring and structures supporting professional work and well-being *Subtheme I* : Temporary and ad hoc *organizational structuring that supports professional work and well-being* which enables the nurses to deal effectively with emerging situations *Subtheme 2* : Long-term *organizational structures that support professional work and well-being,* i.e., persistent work methods, routines, and policies that facilitate the nurses' every-day work and ensure their ability to provide high-quality care
*Theme 2* : Individual recognition and professional acknowledgement *Subtheme 1* : Catering for the social needs of nurses *to be seen and recognized* as unique individuals, with personal needs, wishes and requirements *Subtheme 2* : Professional acknowledgement through the *valuation and utilization of the nurses' specific competences,* also by respecting the boundaries of these competences
